# Biopsychological pattern underlying the psychosomatic symptoms of patients with Hwabyung from a universal perspective

**DOI:** 10.1186/s13030-025-00340-0

**Published:** 2025-10-30

**Authors:** Han Chae, Soo Jin Lee, Seok In Yoon, Hui-Yeong Park, Jong Woo Kim

**Affiliations:** 1 School of Korean Medicine, Pusan National University, Busan, Korea; 2 Department of Psychology, Kyungsung University, Busan, Korea; 3 Department of Neuropsychiatry, College of Korean Medicine, KyungHee University, 892 Dongnam-ro, Gangdong-gu, Seoul, Korea

**Keywords:** Psychosomatic symptoms, Culture-bound syndrome, Hwabyung, Hwabyung test, Sasang personality questionnaire, Eum-Yang psychology

## Abstract

**Background:**

Hwabyung is a psychiatric syndrome originally described in Korea that presents as chronic psychosomatic distress with emotional dysregulation and heightened somatic arousal. However, no objective analysis to clarify its progressive mechanism within a universal biopsychological framework has as yet been done that incorporates insights from traditional East Asian medical psychology.

**Methods:**

We recruited 118 patients with Hwabyung and assessed their psychological and somatic symptoms using the Hwabyung Test (HB). Levels of depression, anxiety, and anger expression, as well as biopsychological features were evaluated with the Sasang Personality Questionnaire (SPQ). Psychological and somatic symptoms of Hwabyung were predicted through a regression analysis that used three SPQ subscales: behavioral activation (SPQ-B), cognitive flexibility (SPQ-C), and emotional responsiveness (SPQ-E). Hwabyung subgroups were identified by K-means analysis and their psychosomatic and biopsychological patterns were analyzed with HB and SPQ through ANCOVA and Profile Analysis to explore underlying biopsychological dimensions beyond culture-specific frameworks.

**Results:**

The SPQ subscales explained 26.0% of the psychological and 14.3% of the somatic symptoms of Hwabyung. Three distinct Hwabyung subgroups (mild, moderate, and severe) were identified based on the severity of psychological and somatic symptoms. Patients with severe symptoms showed a unique SPQ subscale profile with high SPQ-B, low SPQ-C, and low SPQ-E scores, reflecting volatile, aggressive, rigid, pessimistic, repressed, and isolated biopsychological characteristics.

**Discussion:**

This study suggests a representative SPQ subscale profile of Hwabyung and underlying mind-body interaction mechanisms within East-Asian biopsychological theory. It offers a more comprehensive and generalizable understanding of Hwabyung and other culture-bound psychosomatic syndromes, supporting improved diagnostic and intervention strategies across populations.

## Introduction

Hwabyung was first reported among Koreans to occur due to the accumulation of repressed emotional responses to stressful events that should have been properly processed or resolved [1, 2]. Patients with Hwabyung have been reported to experience psychiatric symptoms such as increased irritability or anger, insomnia, Major Depressive Disorder (MDD), and difficulties in interpersonal relationships. In addition, they exhibit typical somatic symptoms, including a sensation of heat, flushing, headache, a lump in the throat and chest, a pushing-up sensation in the chest, and respiratory discomfort characterized by chest tightness and frequent sighing [1, 3, 4, 5].

In Korea, these patients have been treated clinically for hundreds of years, and the official Clinical Guideline for Hwabyung was established in 2013 and revised in 2021 [4, 6]. Its prevalence rate ranges from 4.2% to 13.3%, and it is common among middle-aged women [5]. In 2022, the number of patients with Hwabyung was 11,587 according to the Health Insurance Review and Assessment Service (HIRA). Hwabyung was incorporated in the Diagnostic and Statistical Manual of Mental Disorders (DSM-IV, 1994) as a culture-bound syndrome and classified under the code F48.8 (Other specified nonpsychotic mental disorders) in the International Classification of Diseases (ICD-10, 1990) and U22.2 (Hwabyeong) in the Korean Standard Classification of Diseases (KCD, 2016) [4].

Previous studies have raised questions about whether Hwabyung exists as an independent entity [7, 8], noting its similarities to somatization disorders, MDD, and conversion disorder [2, 4, 9, 10]. Particularly in early research, Korean socio-cultural characteristics [1, 4, 8] such as *Han* (한, 恨) and Confucian culture, which emphasize obedience and conformity to social norms, have been proposed as important etiological factors or mechanisms exacerbating symptoms [5]. Consequently, several clinical symptoms, including depression, anger, and somatic complaints are considered key indicators for diagnosis [11], although there are ongoing debates over its unique pathological features [12].

However, Hwabyung originally refered to a psychosomatic condition traditionally conceptualized as ‘Repressed Fire Disease (울화병, 鬱火病)’ in traditional Korean psychiatry that occurs when so-called *qi* stagnation or psychophysiological blockage due to unresolved stress from emotional repression leads to accumulation of internal heat or heightened physiological arousal, resulting in a chronic complex of psychological and somatic symptoms [3, 5]. As a psychosomatic disorder with a unique pathophysiology, Hwabyung has been treated for centuries using traditional Korean medical interventions distinct from Western approaches [4], such as acupuncture and moxibustion [11, 13], medical herbs [14], and mind-body intervention [15]. These have recently been supported by scientific clinical research that has demonstrated their clinical usefulness [6, 16].

Despite the long history of diagnosis and intervention and the presence of clear symptoms, defining Hwabyung has been challenging [8]. This difficulty is partly because traditional East Asian biopsychology theories based on the concept of Eum-Yang (Yin-Yang) or biopsychosocial polarity have not been used to objectively analyze the characteristics of the disease [4, 17, 18, 19]. If Hwabyung is considered a disorder unique to Koreans or Korean culture, then traditional Korean psychiatry, with its vast amount of clinical experience, might presumably have the most appropriate and systematic answers. However, reliable and objective psychometric measures to simultaneously analyze the psychosomatic characteristics required to describe the clinical experience of traditional Korean psychiatry in diagnosing and treating Hwabyung have not as yet been provided [19, 20].

The Sasang Personality Questionnaire (SPQ) [20, 21] was recently developed as a validated psychometric instrument grounded in traditional Korean medical psychology, designed to assess temperament-related biopsychological traits based on the principle of integrated psychophysiological functioning [22]. The SPQ has been shown to be useful for analyzing the psychophysiological traits of East-Asian Eum-Yang [4, 19], where Yang represents activated or stimulated physical, psychological, and social characteristics and Eum signifies the opposite, being inhibited or suppressed (Table 1). It consists of three subscales for the analysis of specific behavioral attitudes (SPQ-B), cognitive styles (SPQ-C), and emotional reactions (SPQ-E). Although developed within a traditional Korean medical framework, the SPQ’s subscales correspond to widely recognized psychophysiological constructs such as behavioral activation, cognitive flexibility, and affective responsiveness, making it suitable for cross-cultural and global applications.

**Table 1:** Typical adaptive and maladaptive features of low and high Sasang personality questionnaire (SPQ) subscale score profiles

SPQ subscales		Healthy and Adaptive	Disorder or Maladaptive
SPQ-B	High	Active, energetic, proactive, expressive, sociable	**Hyperactive, volatile, aggressive, easily affected, restless**
	Low	Stable, calm, disciplined, focused, introverted	Passive, avoidant, withdrawn, lethargic, disengaged
SPQ-C	High	Adaptable, optimistic, intuitive, cool-headed, creative	Self-centered, haphazard, moody, unpredictable, impulsive
	Low	Systematic, consistent, meticulous, fair, reasonable	**Cranky, pessimistic, rigid, ruminative, stubborn**
SPQ-E	High	Passionate, engaging, warm, empathetic, affectionate	Unstable, irrational, hostile, oversensitive, dependent
	Low	Rational, composed, calm, neutral, logical	**Cold, detached, indifferent, isolated, emotionless**

Sasang Personality Questionnaire, SPQ; SPQ-Behavior, SPQ-B; SPQ-Cognition, SPQ-C; SPQ-Emotion, SPQ-E

Bold represents the typical SPQ profile (high SPQ-B, low SPQ-C, low SPQ-E) of the patients with Hwabyung in the current study

In relatively healthy subjects, the SPQ has facilitated a variety of clinical studies [18] on adaptive and maladaptive cognitive emotion regulation [23] and problematic behaviors of adolescents [24, 25]. Interestingly, it has been reported that clinically diagnosed MDD patients exhibit a distinctive SPQ subscale profile (low SPQ-B and high SPQ-E) [24]. Considering these previous reports, the SPQ would be useful for more objective assessments and analyses of Hwabyung. Moreover, the SPQ provides a valuable framework for investigating how culturally shaped emotional regulation patterns may manifest in somatic and psychological symptoms, thereby contributing to a more generalizable model of psychosomatic disorders.

Therefore, this study was done to measure the psychological and somatic symptoms clinically diagnosed patients with Hwabyung and to analyze them using the SPQ subscale. Regression analysis was done to determine whether the SPQ subscale can explain the psychosomatic symptoms of Hwabyung [22] and K-means analysis and Profile analysis were used to identify the clinical significance of SPQ subscale profiles across existing subgroups of Hwabyung [24].

The current quantitative research might provide clinicians with an objective analysis of Hwabyung symptoms based on East Asian biopsychological mechanisms, thereby explaining the mind–body interactions and the manifestation of psychological and physical symptoms. As a result, it may help establish accurate and effective diagnostic and treatment strategies grounded in a profound understanding of Hwabyung. Beyond its relevance to Korean clinical settings, this approach may offer meaningful insights into the psychophysiological basis of culture-bound syndromes across diverse populations and help integrate traditional frameworks into global health paradigms.

## Materials & methods

### Procedures and participants

The purpose and procedures of this study were explained to patients who agreed to participate with written consent after responding to hospital advertisements. Participants were recruited based on a diagnosis of Hwabyung, confirmed through clinician interviews that used the Hwabyung Diagnostic Interview Schedule (HBDIS) [26]. However, patients with hallucinations (visual or auditory) or delusions were excluded from the study.

The Hwabyung Test (HB) [27], Hwabyung symptom-related clinical measures, and Sasang Personality Questionnaire (SPQ) [21] were administered, and compensation was provided. The clinical data of 118 participants (95 female) were available for analysis. The average age of participants was 49.42 (SD = 14.83), ranging from 19 to 77 years.

The study was conducted in accordance with the guidelines of the Declaration of Helsinki following approval by the institutional review board of KyungHee University Korean Medicine Hospital at Gangdong (KHNMCOH 2022-02-009-010 [2023.4.26]). Recruitment was done from May 16, 2022, to July 11, 2023.

### Clinical measures

#### Hwabyung diagnostic interview schedule (HBDIS)

The HBDIS is a standardized clinical interview [26] that assesses seven main aspects for the clinical diagnosis of Hwabyung: core somatic symptoms, related somatic symptoms, core psychological symptoms, related psychological symptoms, psychosocial dysfunction, presence of related stress, and presence of medical illness.

A previous study showed an agreement between the HBDIS diagnosis and clinician diagnosis of 0.85, with a sensitivity of 0.83 and specificity of 0.88. The one-month test-retest agreement was reported to be 0.82.

#### Hwabyung test (HB)

The HB [27], comprising 31 items rated on a 5-point Likert scale, assesses both the psychological (HB-PSY, 16 items) and somatic (HB-SOM, 15 items) symptoms of Hwabyung. These items represent a collection of Hwabyung-specific symptoms that differentiate individuals with Hwabyung from both healthy individuals and those with depression.

The HB-PSY subscale measures psychological characteristics, including suppression of emotional expression, discomfort in interpersonal relationships, rigidity, a resigned coping style, a victim mentality, and feelings of unfairness, anxiety, depression, and anger. The HB-SOM subscale measures somatic symptoms, including hot flashes, chest tightness, shortness of breath, and a sensation of a lump or a feeling of something rising in the chest or throat. The total HB score is the sum of the two subscale scores.

Cronbach’s alpha for the HB-PSY and HB-SOM were reported as 0.85 and 0.93, respectively, in the previous study [27] and as 0.934 and 0.880, respectively, in the current study.

#### Hwabyung symptom-related clinical measures

##### Beck depression inventory-II (BDI-II)

The BDI-II [28] consists of 21 items rated on a 4-point Likert scale (0 to 3). Higher scores indicate more severe depressive symptoms, categorized as minimal depression (0∼13 points), mild depression (14∼19 points), moderate depression (20∼28 points), and severe depression (29∼63 points). Cronbach’s alpha for BDI-II was reported as 0.94 in a previous study [29] and 0.905 in the current study.

##### Beck anxiety inventory (BAI)

The BAI [30] consists of 21 items rated on a 4-point Likert scale (0 to 3). Higher scores indicate more severe anxiety symptoms, categorized as mild anxiety (22∼26 points), severe anxiety (27∼31 points), and extreme anxiety (32 points and above). Cronbach’s alpha for the BAI was reported as 0.91 in a previous study [30] and 0.961 in the current study.

##### State-trait anger expression inventory (STAXI)

The STAXI [31] is used to analyze experience and expression of anger. It comprises 24 items rated on a 4-point Likert scale (1 to 4). The scale includes subscales for state anger (STAXI-State, 10 items), trait anger (STAXI-Trait, 10 items), and anger expression (24 items), which is further divided into three subscales: anger control (STAXI-Control, 8 items), anger suppression (STAXI-Suppression, 8 items), and anger expression (STAXI-Expression, 8 items). Higher scores indicate greater frequency and intensity of anger experiences and expressions.

Cronbach’s alpha for the STAXI-State, STAXI-Trait, STAXI-Control, STAXI-Suppression, and STAXI-Expression were reported as 0.90, 0.75, 0.79, 0.66, and 0.70, respectively, in a previous study [31] and as 0.957, 0.873, 0.783, 0.837, and 0.828, respectively, in the current study.

##### Eogul scale (ES)

Eogul is a culturally specific psychopathological concept in Korea that is associated with Hwabyung and characterized by pent-up frustration and resentment from feelings of unfairness and suppression of negative emotional responses. The Eogul scale (ES) [17] is used to assess the level of Eogul and consists of 15 items rated on a 5-point Likert scale (0 to 4). The scale includes subscales for Emotional/Physical Reactions (ES-EPR, 6 items), Unjust Beliefs (ES-UB, 6 items), and Avoidant Behaviors (ES-AB, 3 items). The total ES score is the sum of the three subscale scores. Higher scores indicate greater severity and frequency of Eogul experiences and expressions.

Cronbach’s alpha for the ES total, ES-EPR, ES-UB, and ES-AB were reported as 0.91, 0.91, 0.86, and 0.80, respectively, in a previous study [17] and as 0.958, 0.899, 0.909, and 0.820, respectively, in the current study.

#### Sasang personality questionnaire (SPQ)

The SPQ, consisting of 20 items rated on a 4-point Likert scale, is a multidimensional biopsychological test that measures Eum-Yang biopsychological characteristics in both healthy individuals and patients [19, 21]. It comprises three subscales: behavioral attitudes (SPQ-B), cognitive styles (SPQ-C), and emotional reactions (SPQ-E). The SPQ-T is the sum of these (Table 1).

SPQ-B measures the degree of sociability, extraversion, cooperativeness, activeness, vitality, and diligence in behavioral attitudes, while SPQ-C assesses adaptability, spontaneity, independence, straightforwardness, and confidence in cognitive styles. Similarly, SPQ-E measures emotional sensitivity, empathy, emotional intensity, passion, and anxiety in emotional reactions. A high score on the SPQ-T reflects an activated or stimulated biopsychosocial disposition, while a low score represents an inhibited or suppressed disposition that is conceptually aligned with Yang-like and Eum-like characteristics, respectively.

Scores on each SPQ subscale reflect unique psychological characteristics [19]; a subscale may indicate both healthy (adaptive) and disordered (maladaptive) behaviors depending on an individual’s response to stressful situations and overall psychological health (Table 1). The psychometric structure of the SPQ has been examined with the Temperament and Character Inventory, the Junior Temperament and Character Inventory, the NEO Personality Inventory-Revised, the Behavioral Inhibition System/Behavioral Activation System (BIS/BAS) Scale, the Eysenck Personality Questionnaire, the Positive Affect and Negative Affect Schedule, the Cognitive Emotion Regulation Questionnaire, the Child Behavior Checklist, the Youth Self-Report, and the Short Form-12 [18, 21, 24].

Previous studies have reported internal consistency coefficients for SPQ-T, SPQ-B, SPQ-C, and SPQ-E in adults of 0.704, 0.861, 0.685, and 0.709, respectively. The test-retest reliability over five months among high school students for SPQ-T, SPQ-B, SPQ-C, and SPQ-E was 0.763, 0.766, 0.727, and 0.704, respectively.

### Statistical analysis

Pearson’s correlation analysis was conducted to examine the relationship between the clinical indices of the HB and the SPQ subscales. Regression analysis with sex and age as covariates was used to predict Hwabyung psychological (HB-PSY) and somatic (HB-SOM) symptoms using three SPQ subscales.

K-means analysis was performed using responses to the 31 items of the Hwabyung Test from Hwabyung patients to identify latent subgroups. K-means analysis is an unsupervised machine learning algorithm employed to partition the data into distinct clusters based on the similarity of their features. This method aims to minimize the within-cluster variance and maximize the between-cluster variance, effectively grouping similar data points together.

The elbow method, based on the Within-Cluster Sum of Squares (WCSS), and the gap statistic were used to determine the optimal number of subgroups. These methods provide a data-driven approach to identify the point of diminishing returns in cluster separation, thus indicating the appropriate number of clusters that best represent the underlying structure of the data.

The significance of differences in HB and SPQ subscale scores between the acquired Hwabyung subgroups was attested using Analysis of Covariance (ANCOVA), with sex and age as covariates. Profile Analysis (PA), including test of parallelism and flatness, was also performed to examine significant differences in HB and SPQ subscale profiles between the acquired Hwabyung subgroups.

All statistical analyses were performed using jamovi 2.6.15 (The jamovi project, https://www.jamovi.org). The results are presented as mean ± standard error or frequency (%). Statistical significance levels were set at *p* < 0.05, *p* < 0.01, and *p* < 0.001.

## Results

### Correlation between Hwabyung symptoms and biopsychological measures

There was a significant correlation between HB and SPQ subscales as shown in Table 2. HB-PSY showed a significant positive correlation with HB-SOM (*r* = 0.764, *p* < 0.001) and HB total (*r* = 0.928, *p* < 0.001) and a negative correlation with SPQ-C (*r*=-0.337, *p* < 0.001), SPQ-E (*r*=-0.302, *p* < 0.001), and SPQ total (*r*=-0.273, *p* < 0.01). HB-SOM exhibited a significant positive correlation with HB Total (*r* = 0.949, *p* < 0.001) and SPQ-B (*r* = 0.241, *p* < 0.01). HB total showed a significant positive correlation with SPQ-B (*r* = 0.216, *p* < 0.05) and a negative correlation with SPQ-C (*r*=-0.261, *p* < 0.01) and SPQ-E (*r*=-0.221, *p* < 0.05).

**Table 2:** Pearson’s correlation coefficients for Hwabyung-related clinical symptoms and SPQ subscales

	HB-PSY	HB-SOM	HB total	SPQ-B	SPQ-C	SPQ-E	SPQ total
HB-PSY	-	0.764***	0.928***	0.157	-0.337***	-0.302***	-0.273**
HB-SOM	0.764***	-	0.949***	0.241**	-0.168	-0.127	-0.051
HB total	0.928***	0.949***	-	0.216*	-0.261**	-0.221*	-0.162
BDI-II	0.569***	0.651***	0.569***	0.249**	-0.168	-0.124	-0.055
BAI	0.606***	0.746***	0.606***	0.197*	-0.151	-0.081	-0.024
STAXIS-State	0.492***	0.696***	0.492***	0.143	-0.143	-0.024	-0.011
STAXIS-Trait	0.400***	0.605***	0.400***	0.405***	-0.085	-0.047	0.112
STAXIS-Control	0.306***	0.122	0.306***	-0.156	-0.416***	0.012	-0.252**
STAXIS-Suppression	0.656***	0.639***	0.656***	0.197*	-0.151	-0.222*	-0.132
STAXIS-Expression	0.203*	0.393***	0.203*	0.44***	-0.006	0.216*	0.307***
ES total	0.673***	0.817***	0.799***	0.215*	-0.276**	-0.115	-0.106

*, *p* < 0.05; **, *p* < 0.01; ***, *p* < 0.001

Hwabyung Test, HB; HB-Psychological symptoms, HB-PSY; HB-somatic symptoms, HB-SOM; Beck Depression Inventory-II, BDI-II; Beck Anxiety Inventory, BAI; State Trait Anger Inventory, STAXI; Eogul Scale, ES; Sasang Personality Questionnaire, SPQ; SPQ-Behavior, SPQ-B; SPQ-Cognition, SPQ-C; SPQ-Emotion, SPQ-E

Table 2 presents the correlation coefficients for Hwabyung-related clinical symptoms, including those assessed by the Beck Depression Inventory-II (BDI), Beck Anxiety Inventory (BAI), State-Trait Anger Expression Inventory (STAXI), Eogul Scale (ES), and SPQ subscales.

### Prediction of Hwabyung psychological and somatic symptoms using SPQ subscales

Regression analysis with sex and age as covariates was conducted to explain the psychological and somatic symptoms of Hwabyung, as shown in Table 3.

**Table 3:** Regression analysis using SPQ subscales with age and sex as covariates on Hwabyung psychological and somatic symptoms

	Unstandardized coefficient		Standardized coefficient	t	*p*
	B	SE	Β		
HB-PSY					
SPQ-B**	0.967	0.299	0.273	3.23	0.002
SPQ-C***	-1.168	0.324	-0.290	-3.607	< 0.001
SPQ-E***	-1.054	0.24	-0.371	-4.382	< 0.001
Sex*	4.344	1.927	0.462	2.255	0.026
Age	0.058	0.052	0.091	1.123	0.264
F(5,112) = 9.231, p = < 0.001, *R* = 0.540 (adj.R²=0.260)					
HB-SOM					
SPQ-B***	1.333	0.381	0.319	3.497	< 0.001
SPQ-C	-0.665	0.412	-0.139	-1.613	0.110
SPQ-E*	-0.762	0.306	-0.227	-2.49	0.014
Sex	4.777	2.453	0.43	1.948	0.054
Age*	0.141	0.066	0.189	2.153	0.033
F(5,112) = 4.912, *p* < 0.001, *R* = 0.424 (adj.R²=0.143)					
HB total					
SPQ-B***	2.3	0.635	0.317	3.624	< 0.001
SPQ-C**	-1.833	0.687	-0.222	-2.67	0.009
SPQ-E***	-1.816	0.51	-0.312	-3.562	< 0.001
Sex*	9.121	4.085	0.473	2.233	0.028
Age	0.199	0.109	0.153	1.822	0.071
F(5,112) = 7.209, p = < 0.001, *R* = 0.493 (adj.R²=0.210)					

*, *p* < 0.05; **, *p* < 0.01; ***, *p* < 0.001

Hwabyung Test, HB; HB-Psychological symptoms, HB-PSY; HB-somatic symptoms, HB-SOM; Sasang Personality Questionnaire, SPQ; SPQ-Behavior, SPQ-B; SPQ-Cognition, SPQ-C; SPQ-Emotion, SPQ-E

A significant model explained the psychological symptoms of Hwabyung and accounted for 26.0% of the variance (F(5,112) = 9.231, *p* < 0.001), with SPQ-B (ß=0.273, *p* = 0.002), SPQ-C (ß=-0.290, *p* < 0.001), SPQ-E (ß=-0.371, *p* < 0.001), and sex (ß=0.462, *p* = 0.026) significant.

A significant model explained the somatic symptoms of Hwabyung and accounted for 14.3% of the variance (F(5,112) = 4.912, *p* < 0.001), with SPQ-B (ß=0.312, *p* = 0.001), SPQ-E (ß=-0.215, *p* = 0.023), and age (ß=0.189, *p* = 0.033) significant.

A significant model explained the Hwabyung symptoms and accounted for 21.0% of the variance (F(5,112) = 7.209, *p* < 0.001), with SPQ-B (ß=0.317, *p* < 0.001), SPQ-C (ß=-0.222, *p* = 0.009), SPQ-E (ß=-0.312, *p* = 0.001), and sex (ß=0.473, *p* = 0.028) significant.

### Clinical features of the mild, moderate, and severe Hwabyung subgroups

**Fig. 1:**
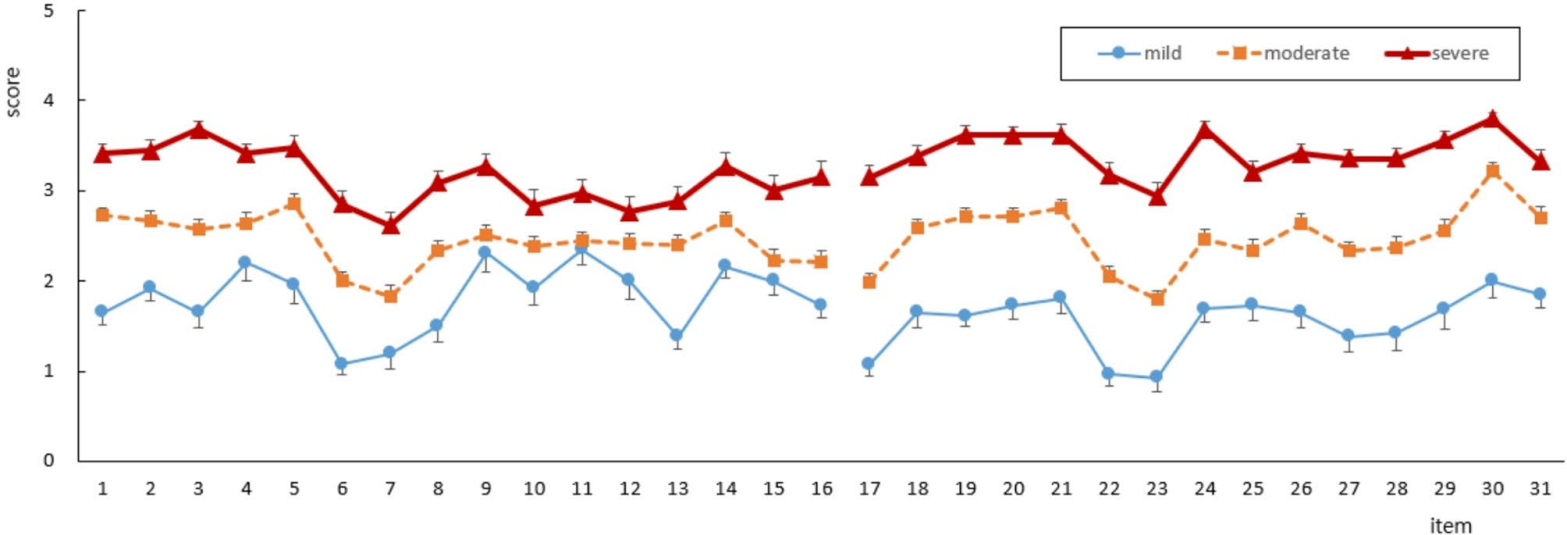
Item scores for the psychological (item 1–16) and somatic (item 17–31) symptoms of three Hwabyung subgroups extracted using K-means analysis

Three distinct subgroups (mild, moderate, and severe) were identified using K-means analysis based on the severity of their Hwabyung psychological and somatic symptoms (Fig. 1). The optimal number of subgroups was determined by identifying the point where the decrease in the WCSS sharply declined, coinciding with the largest gap statistic value and the smallest number of clusters.

**Fig. 2:**
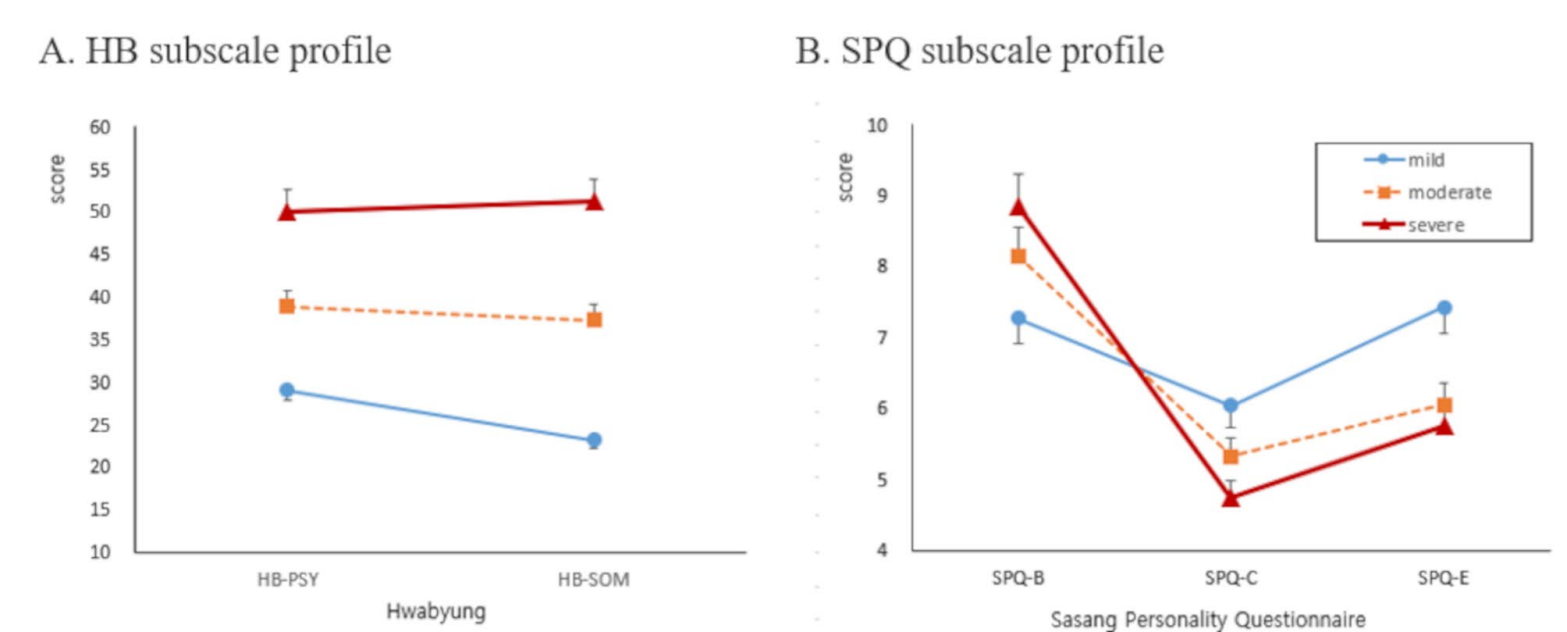
HB and SPQ subscales profiles of the mild, moderate, and severe Hwabyung subgroups

Distinctive differences in HB and SPQ subscales among the three Hwabyung subgroups were examined using ANCOVA and Profile Analysis, as shown in Table 4; Fig. 2.

**Table 4:** Estimated clinical features of HB and SPQ subscales in mild, moderate, and severe Hwabyung subgroups with age and sex as covariates

Measure	Subscales	mild (*n* = 26)	moderate (*n* = 58)	severe (= 34)	Statistical analysis	
HB						
	HB-PSY***	28.66 ± 1.14	38.31 ± 0.85	49.41 ± 1.10	F = 98.612, *p* < 0.001	mild < moderate < severe
	HB-SOM***	23.04 ± 0.98	36.92 ± 0.74	50.73 ± 0.95	F = 231.856, *p* < 0.001	mild < moderate < severe
	HB total***	51.70 ± 1.62	75.23 ± 1.21	100.14 ± 1.57	F = 262.151, *p* < 0.001	mild < moderate < severe
BDI-II***		12.72 ± 1.66	22.58 ± 1.24	33.78 ± 1.61	F = 36.716, *p* < 0.001	mild < moderate < severe
BAI***		12.52 ± 2.03	21.76 ± 1.52	39.42 ± 1.97	F = 56.118, *p* < 0.001	mild < moderate < severe
STAXI						
	STAXIS-State***	15.05 ± 1.28	20.13 ± 0.96	29.24 ± 1.24	F = 38.602, *p* < 0.001	mild < moderate < severe
	STAXIS-Trait***	22.10 ± 1.02	25.38 ± 0.77	31.64 ± 0.99	F = 27.678, *p* < 0.001	mild < moderate < severe
	STAXIS-Control	18.76 ± 0.76	18.73 ± 0.57	19.56 ± 0.73	F = 0.568, *p* = 0.568	
	STAXIS-Suppression***	16.80 ± 0.72	19.98 ± 0.54	25.84 ± 0.70	F = 49.797, *p* < 0.001	mild < moderate < severe
	STAXIS-Expression***	15.16 ± 0.86	17.56 ± 0.64	19.98 ± 0.83	F = 9.214, *p* < 0.001	mild, moderate < severe
ES total***		17.76 ± 1.83	30.32 ± 1.37	45.54 ± 1.77	F = 68.554, *p* < 0.001	mild < moderate < severe
SPQ						
	SPQ-B	7.23 ± 0.53	8.10 ± 0.40	8.80 ± 0.51	F = 2.545, *p* = 0.083	
	SPQ-C	6.11 ± 0.47	5.44 ± 0.35	4.87 ± 0.45	F = 2.054, *p* = 0.133	
	SPQ-E	7.32 ± 0.66	5.88 ± 0.50	5.54 ± 0.64	F = 2.333, *p* = 0.102	
	SPQ total	20.67 ± 1.08	19.43 ± 0.81	18.93 ± 1.05	F = 0.775, *p* = 0.463	

***, *p* < 0.001

Hwabyung Test, HB; HB-Psychological symptoms, HB-PSY; HB-somatic symptoms, HB-SOM; Beck Depression Inventory-II, BDI-II; Beck Anxiety Inventory, BAI; State Trait Anger Inventory, STAXI; Eogul, ES; Sasang Personality Questionnaire, SPQ; SPQ-Behavior, SPQ-B; SPQ-Cognition, SPQ-C; SPQ-Emotion, SPQ-E

There were significant differences in estimated HB-PSY (F = 98.612, *p* < 0.001), HB-SOM (F = 231.856, *p* < 0.001), and HB total (F = 262.151, *p* < 0.001) scores among the three subgroups (Table 4). The HB-PSY, HB-SOM, and HB total scores of the mild, moderate, and severe subgroups increased in that order, with each subgroup exhibiting statistically significant differences from the others. However, there were no significant differences in estimated SPQ subscale scores among these three subgroups. The BDI-II, BAI, STAXI subscales (except for STAXI-control), as well as ES total scores, showed an increasing order across the mild, moderate, and severe subgroups, with each subgroup exhibiting statistically significant differences from the others (Table 4).

The HB and SPQ subscale profiles showed distinct differences among the mild, moderate, and severe Hwabyung subgroups (Fig. 2). The profile of the HB subscale, encompassing HB-Psychological and HB-Somatic symptoms, was not flat (flatness, df = 1, F = 10.105, *p* = 0.002). The parallelism of the HB subscale profile with the interaction of the Hwabyung subgroup were significantly different (parallelism, df = 2, F = 7.476, *p* < 0.001).

The profile of the SPQ subscales (SPQ-B, SPQ-C, and SPQ-E) was not flat (flatness, df = 2, F = 31.773, *p* < 0.001). Furthermore, the parallelism of the SPQ subscale profile with the interaction of Hwabyung subgroup were significantly different (parallelism, df = 4, F = 3.855, *p* = 0.005).

## Discussion

In this study, we analyzed the Eum-Yang biopsychological pattern of the psychological and somatic symptoms of Hwabyung patients to explain their psychosomatic symptoms using regression analysis, identify a distinct SPQ subscale profile using K-means and profile analysis, and to explain underlying mind-body mechanisms within the framework of traditional Korean psychiatry.

First, a high SPQ-B score reflects activated or stimulated (Yang) behaviors (e.g., extraversion, activeness) and symptomatic expressions [21]. SPQ-B explained the somatic (ß=0.319, *p* < 0.001) and psychological symptoms (ß=0.273, *p* < 0.001) of Hwabyung. SPQ-B was positively correlated with somatic symptoms of Hwabyung (*r* = 0.241), trait anger (*r* = 0.405), and external expression of anger (*r* = 0.440).

These findings indicate that a higher SPQ-B is associated with psychological manifestations such as symptoms of depression, anxiety, and anger, which involve the intermittent externalization of negative psychological energy (Tables 2 and 3). It may also be associated with heightened or stimulated (‘Fire’) somatic symptoms, including palpitations, heat sensations, flushing, headaches, a pushing-up sensation in the chest, respiratory discomfort such as chest tightness, and frequent sighing [5].

Second, a low SPQ-C score represents a repressive or inhibitory (Eum) cognitive style (e.g., inflexibility, conservatism) and a low SPQ-E score indicates inhibited or suppressed (Eum) emotional responses (e.g., emotional insensitivity, apathy) [21]. SPQ-C specifically explained the psychological symptoms of Hwabyung (ß=-0.290, *p* < 0.001) and was negatively correlated with the its psychological symptoms (*r*=-0.337) and anger control (*r*=-0.416). Moreover, SPQ-E explained the psychological (ß=-0.371, *p* < 0.001) and somatic symptoms (ß=-0.227, *p* < 0.05) of Hwabyung and showed a negative correlation with the psychological symptoms of Hwabyung (*r*=-0.302).

These findings indicate that a lower SPQ-C score is associated with the psychological aggravation of Hwabyung, along with heightened anger suppression and feelings of unfairness. A lower SPQ-E score correspond to more severe levels of both psychological and somatic symptoms of Hwabyung, along with heightened anger suppression and reduced anger expression (Tables 2 and 3).

The SPQ-C plays a role in recognizing, analyzing, and controlling responses to external stress [5, 21]. Therefore, rather than resolving external stress in a bold, adaptive, and positive manner (as illustrated on the left side of Table 1), patients with Hwabyung tend to fixate on minor issues and exhibit psychopathological thinking characterized by crankiness, pessimism, rigidity, rumination, and stubbornness (as shown on the right side of Table 1) [4, 5]. The SPQ-E plays a role in expressing one’s emotional state appropriately and constructively, facilitating healthy emotional communication with others while maintaining empathic connections. Therefore, rather than expressing emotions in a balanced manner fostering meaningful interpersonal connections (as illustrated on the left side of Table 1), patients with Hwabyung tend to exhibit emotional dysregulation, alternating between withdrawal and inappropriate outbursts, leading to interpersonal difficulties (as shown on the right side of Table 1),

Consequently, a low SPQ-C score appears to reflect a psychological tendency to internalize stress, somatize it, and suppress anger, while a low SPQ-E score is associated with emotional dysregulation, resulting in both suppression and explosive outburst of anger, thereby aggravating psychological and somatic symptoms.

Lastly, this study aimed to identify key biopsychological factors that explain the psychosomatic symptoms of Hwabyung by employing regression analysis and ANOVA. In addition, we sought to delineate a characteristic Eum-Yang subscale profile of Hwabyung that distinguishes it from other psychological disorders in a person-centered approach [32].

The current study showed that the SPQ subscales were able to explain 26.0% of the psychological and 14.3% of the somatic symptoms of Hwabyung (Table 3). The somatic symptoms of Hwabyung were associated with high SPQ-B scores and worsened with increasing age, while the psychological symptoms were associated with low SPQ-C and SPQ-E scores and were more severe in women (Tables 2 and 3).

From a person-centered perspective, the Hwabyung symptoms were exacerbated in individuals with an HLL SPQ subscale profile, which is characterized by high SPQ-B (behavioral irritability and impulsivity), low SPQ-C (cognitive rigidity and pessimism), and low SPQ-E (emotional isolation and vulnerability), with greater severity observed in women (Table 4; Fig. 2). The etiology and pathological mechanism of Hwabyung might be explained from the perspective of integrated psychophysiological functioning grounded in an East-Asian perspective, using the distinct HLL SPQ subscale profile (high SPQ-B, low SPQ-C, and low SPQ-E).

In traditional Eastern societies of collectivistic cultures that value social harmony over personal autonomy and individualism [32], individuals are often unable to express emotional responses to stressful situations directly, leading to the internalization of unrelieved emotions [5]. In the Korean socio-cultural context, the concept of *Han* (한, 恨) is commonly described as the long-term accumulation and internalization of sorrow and unresolved emotional stress. It has been used to explain depressive states driven by chronic frustration and repressed anger, which may eventually manifest as intermittent emotional outbursts [3, 5, 16].

The current study suggests that the combination of a biopsychological Eum-Yang profile (high SPQ-B, low SPQ-C, and low SPQ-E) and a collectivistic culture suppressing emotional responses may act synergistically to exacerbate the development and severity of Hwabyung. This finding may provide a rationale for Hwabyung to be recognized globally as a distinct and independent medical entity.

These findings with SPQ subscales present interesting clinical insights from three perspectives.

First, patients with Hwabyung have been reported to exhibit behaviors such as suppression-inhibition-withdrawal, avoidance of stimulus and tension, externalization, and impulsiveness in their defense mechanisms and coping strategies [33]. The HLL SPQ subscale profile of Hwabyung might explain these aspects as a coherent complex of characteristics in which suppression-inhibition-withdrawal corresponds to low SPQ-C, the avoidance of stimulus and tension to low SPQ-E, and both externalization and impulsiveness to high SPQ-B.

Second, the patients of this study with Hwabyung showed a HLL SPQ subscale profile (high SPQ-B, low SPQ-C, and low SPQ-E), which represents characteristics distinctly different from those found in previous study of MDD with an L*H SPQ subscale profile [24]. MDD, which emphasizes a state of being depressed or emotionally low, is characterized by low SPQ-B (avoidant, lethargic, and disengaged behavior) and high SPQ-E (overwhelmed by unstable and oversensitive emotions), accompanied by a low appetite and diminished interest in food [24]. In contrast, Hwabyung is identified by high SPQ-B (irritable, aggressive, volatile), low SPQ-C (ruminative, obsessive, stubborn), and low SPQ-E (unexpressed/cold, socially isolated), along with unique somatic symptoms and emotional repression [19, 21].

Third, the difference in the SPQ subscale profiles of the mild (MMM) and severe (LHH) states of Hwabyung suggests directions for psychological interventions (Fig. 2). Based on the SPQ subscale profile (as shown on the right and left side of Table 1), it might be understood that for the severe symptoms of Hwabyung patients to shift towards a milder state, the SPQ-B score must decrease (becoming more stable and less aggressive), the SPQ-C score must increase (becoming more flexible and positive), and the SPQ-E must also increase (enhancing empathy and interaction with others) [19, 21]. This might be seen as providing a major direction for the psychological intervention of Hwabyung.

Based on these perspectives, a three-stage progression of Hwabyung, or Repressed Fire Disease, which may initially appear to be a collection of unrelated complex symptoms, can be proposed, explaining the mind–body interactions and the manifestation of psychological and physical symptoms throughout its course.

Stage One involves emotional repression, where prolonged suppression of negative emotions leads to psychophysiological blockage [3, 5]. This pattern appears frequently and significantly within Eastern socio-cultural contexts, where emotional restraint is traditionally emphasized. Stage Two consists of the somatized symptoms prominently reported in early Hwabyung studies [1], such as heat sensations, chest stuffiness, and a lump-like feeling rising in the chest [2, 3, 10], typically accompanied by depressive states. Stage Three, increasingly highlighted in recent studies [2, 4, 5], refers to eruptive anger responses that occur when unresolved psychological stress (e.g., a sense of unfairness or Eogul) and accumulated suppressed emotions surpass a threshold of control, resulting in outbursts triggered by relatively minor stimuli.

However, in light of the Korean traditional medical theory that *qi* stagnation (i.e., psychophysical blockage) generates internal heat (i.e., heightened physiological arousal), which manifests as both somatic and psychological (anger) symptoms [3, 5], it is conceivable that Stages Two and Three may not represent a temporal sequence, but rather two distinct subtypes: a somatic-dominant subtype and an psychological-dominant subtype within the same pathological process. Therefore, future studies should explore whether these symptoms appear independently, sequentially, or in combination across patients, and investigate how SPQ subscales that reflect behavioral, cognitive, and emotional dimensions are related to these varied clinical presentations.

These findings, while informed by East Asian concepts of biopsychosocial polarity, highlight pathophysiological dynamics that may transcend cultural boundaries and reflect common mechanisms of psychosomatic distress. Our study demonstrates that the clinical profile of Hwabyung uniquely exhibits an HLL SPQ subscale profile, which might help recognize its universality. This approach may facilitate a deeper understanding of the pathological mechanisms, which would enable clinicians to provide accurate diagnoses and develop effective treatment strategies [4, 5].

However, there are certain limitations in generalizing the findings presented in this study, and further research is needed to address these issues. First, this is a cross-sectional study conducted solely with Hwabyung patients. Therefore, the findings should be validated through clinical research comparing this group with healthy controls and patients diagnosed with MDD and somatic and conversion disorders [5, 10]. Further longitudinal research with a larger, more diverse population is needed to observe actual changes in SPQ subscale scores following treatment or disease progression. Additionally, to determine whether these findings are unique to Koreans or Korean culture or have broad applicability, replication studies involving foreign residents of Korea and Koreans living abroad are needed.

Second, beyond the HBDIS, HB, and SPQ used in this study, other clinical assessment tools [5, 10] along with various assessments of psychological and physical symptoms should be employed to further evaluate the characteristic clinical features of patients with Hwabyung. This would enhance the validity of our findings and help establish Hwabyung as a distinct disorder with a therapeutic framework rather than as a vague culture-bound syndrome [6, 8].

Third, this study presented Hwabyung as having universal psychosomatic symptoms and pathophysiological mechanisms based on clinical assessment informed by a theoretical construct originating from traditional Korean psychology [18, 19]. Through this biopsychological lens, Hwabyung may serve as a prototype for exploring culture-linked expressions of psychological distress that manifest somatically, providing a bridge between culturally specific symptomatology and generalizable diagnostic models.

Future studies should examine whether this research methodology applies to culturally, ethically, or regionally bound syndromes, such as Amok (Philippines), Tatari-byo (Japan), Dhat Syndrome (India), and Ataque de Nervis (Latin America). Such investigations could bridge the gap between emic approaches, which emphasize cultural specificity, and etic approaches, which seek comprehensive diagnostic concepts and criteria.

## Conclusions

This study identified a typical HLL SPQ subscale profile and progressive mechanisms of Hwabyung within a clear biopsychological framework inspired by East Asian theories. It is our hope that this study will contribute to a more comprehensive understanding of Hwabyung and support more effective diagnostic and intervention strategies. Furthermore, the integration of SPQ-based assessment with modern biopsychological paradigms may contribute to the development of transdiagnostic tools applicable across culturally diverse psychosomatic syndromes.

## Data Availability

Raw data is available upon reasonable request.
